# Jeju Magma-Seawater Inhibits α-MSH-Induced Melanogenesis via CaMKKβ-AMPK Signaling Pathways in B16F10 Melanoma Cells

**DOI:** 10.3390/md18090473

**Published:** 2020-09-18

**Authors:** Minhyeok Song, Jihyun Lee, Young-Joo Kim, Dang Hieu Hoang, Wonchae Choe, Insug Kang, Sung Soo Kim, Joohun Ha

**Affiliations:** 1 Department of Biochemistry and Molecular Biology, Graduate School, College of Medicine, Kyung Hee University, 26, Kyungheedae-ro, Dongdaemun-gu, Seoul 02447, Korea; thdalsgur77@khu.ac.kr (M.S.); hoang.dang.hieu.ls@gmail.com (D.H.H.); wchoe@khu.ac.kr (W.C.); iskang@khu.ac.kr (I.K.); sgskim@khu.ac.kr (S.S.K.); 2 Easy Hydrogen Corporation, Jeju City 63196, Korea; easyhydrogen@gmail.com; 3 Department of Urology, College of Medicine, Jeju National University, Jeju City 63196, Korea; kurology@jejunu.ac.kr

**Keywords:** Jeju magma-seawater, melanogenesis, AMPK, PKA, MAPK

## Abstract

Melanin protects skin from ultraviolet radiation, toxic drugs, and chemicals. Its synthesis is sophisticatedly regulated by multiple mechanisms, including transcriptional and enzymatic controls. However, uncontrolled excessive production of melanin can cause serious dermatological disorders, such as freckles, melasma, solar lentigo, and cancer. Moreover, melanogenesis disorders are also linked to neurodegenerative diseases. Therefore, there is a huge demand for safer and more potent inhibitors of melanogenesis. In the present study, we report novel inhibitory effects of Jeju magma-seawater (JMS) on melanogenesis induced by α-melanocyte stimulating hormone (α-MSH) in B16F10 melanoma cells. JMS is the abundant underground seawater found in Jeju Island, a volcanic island of Korea. Research into the physiological effects of JMS is rapidly increasing due to its high contents of various minerals that are essential to human health. However, little is known about the effects of JMS on melanogenesis. Here, we demonstrate that JMS safely and effectively inhibits α-MSH-induced melanogenesis via the CaMKKβ (calcium/calmodulin-dependent protein kinase β)-AMPK (5′ adenosine monophosphate-activated protein kinase) signaling pathway. We further demonstrate that AMPK inhibits the signaling pathways of protein kinase A and MAPKs (mitogen-activated protein kinase), which are critical for melanogenesis-related gene expression. Our results highlight the potential of JMS as a novel therapeutic agent for ameliorating skin pigmentation-related disorders.

## 1. Introduction

Melanin is a darkly pigmented biopolymer that represents the primary color determinant for the skin, hair, and eyes, and it also protects skin from UV radiation, toxic drugs, and chemicals [1]. Melanin is synthesized in the melanosome (a lysosome-related organelle of melanocytes); this occurs through a highly organized process known as melanogenesis, which is tightly regulated by multiple mechanisms including transcriptional and enzymatic control [2]. Tyrosinase, tyrosinase-related protein 1 (Trp1), and tyrosinase-related protein 2 (Trp2) are three key enzymes involved in transforming tyrosine to melanin pigments, and these enzymes are transcriptionally regulated by the microphthalmia-associated transcription factor (MITF) [3]. The paracrine relationship between melanocytes and keratinocytes is critical for UV-induced melanogenesis [4]. Upon exposure to UV radiation, keratinocytes secrete various cytokines and growth factors, including α-melanocyte stimulating hormone (α-MSH). α-MSH then binds to the melanocortin 1 receptor (MC1R) present in the melanocyte cell membrane and triggers several signaling pathways to induce melanogenesis [4]. It has been well established that α-MSH increases the intracellular concentration of cAMP (Cyclic adenosine monophosphate), which results in the activation of protein kinase A (PKA) [5]. PKA in turn phosphorylates the CREB (cAMP response element-binding protein), which is a transcription factor for many genes, including MITF [5]. In addition, MAPKs (mitogen-activated protein kinase) including ERK (extracellular signal-regulated kinase), p38, and JNK (Jun N-terminal kinase) have also been implicated in the up-regulation of melanogenesis in response to α-MSH [6,7,8,9,10,11]. As such, melanin synthesis is sophisticatedly regulated by multiple mechanisms. However, uncontrolled excessive production of melanin can cause serious dermatological disorders, such as freckles, solar lentigo, melasma, and cancer [12,13,14]. Moreover, recent studies have suggested that several melanogenesis disorders are associated with neurodegenerative diseases such as Alzheimer’s, Parkinson’s, and Huntington’s diseases [15,16,17,18,19]. Therefore, there is a huge need to develop safer and more potent inhibitors of melanogenesis.

Deep-sea water, which is pumped up from a depth of more than 500~600 m in various regions of the deep sea, contains high levels of minerals that are known to be essential to human health, including, Ca, Cl, Fe, Na, Mg, K, Se, V, and Zn. Accumulating evidence shows that deep-sea water has beneficial effects on cardiovascular disease, diabetes, obesity, and cancer [20,21,22]. This is not entirely surprising, given that metabolic disorders such as diabetes are associated with significant mineral deficiencies and imbalances [23]. Deep-sea water has been recognized as a safe, natural resource in the fields of cosmetics, food, agriculture, and medicine in several countries, including Korea, Japan, Taiwan, China, and the USA. Jeju Island is a volcanic island located in the southern part of Korea; at its northern region, it boasts a huge pool of underground seawater that was formed through natural filtration of seawater through porous basaltic rocks [24,25,26]. This water is called “Jeju magma-seawater” (herein called ”JMS”) and its mineral contents are similar to those of deep-sea water [24,25,26]. JMS offers an economic advantage over deep-sea water because it is collected about 100~200 m underground [24,25,26]. 

Although research on the application of deep-sea water or JMS is rapidly increasing, its effects on melanogenesis have not yet been reported. In the present study, we demonstrate that JMS effectively inhibits melanin synthesis, tyrosinase activity, and melanogenesis-related protein expression levels through the CaMMKβ-AMPK (calcium/calmodulin-dependent protein kinase β-5′ adenosine monophosphate-activated protein kinase) pathway in B16F10 melanoma cells. AMPK plays a central role in coordinating the activity of metabolic pathways in response to cellular energy status and has been recognized as a drug target for metabolic diseases [27,28,29]. AMPK undergoes allosteric regulation by ATP, ADP, and AMP [30,31], and it is also activated by the upstream kinases, LKB1 and CaMKKβ [32]. Thus, JMS may have value as a novel therapeutic agent for ameliorating skin pigmentation-related disorders.

## 2. Results

### 2.1. JMS Inhibits Melanogenesis in B16F10 Melanoma Cells

**Figure 1:**
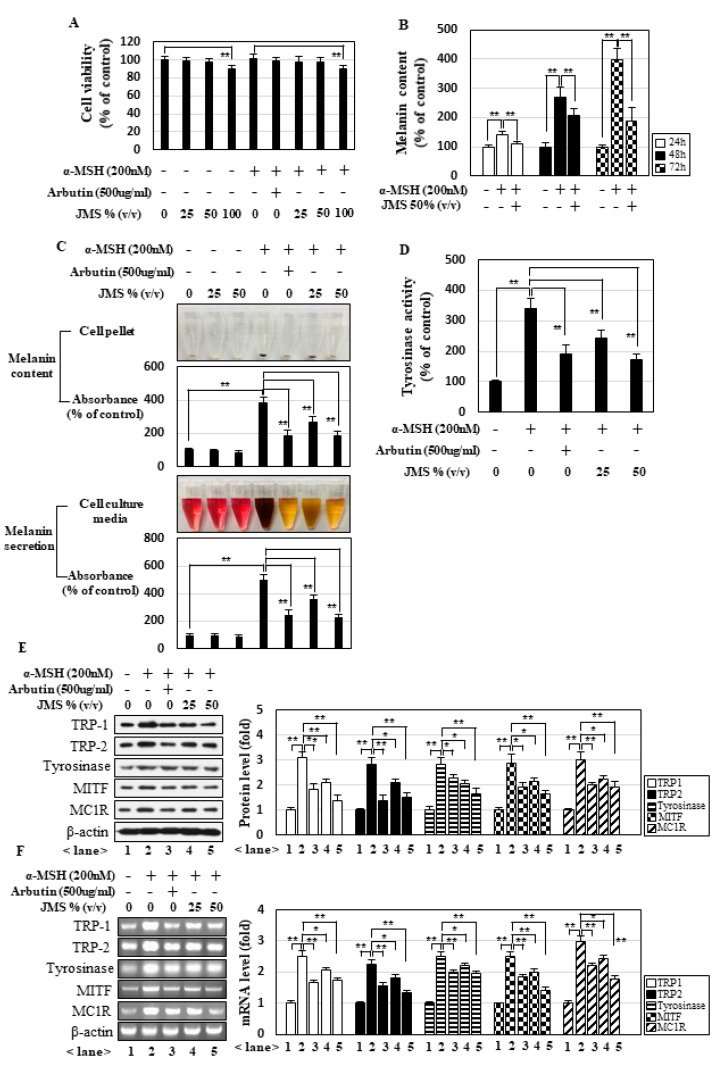
Jeju magma-seawater (JMS) inhibits α-melanocyte stimulating hormone (α-MSH)-induced melanogenesis in B16F10 cells. (**A**) B16F10 cells were incubated for 72 h in Dulbecco’s modified Eagle’s medium (DMEM) made with the indicated concentration of JMS (*v/v*) in the presence or absence of α-MSH, and then the MTT ((3-(4,5-dimethylthiazol-2-yl)-2,5-diphenyl tetrazolium bromide) assay was performed. (**B**) B16F10 cells were treated with 50% JMS for the indicated time periods, and melanin contents were measured. (**C**) After cells were cultured with α-MSH and JMS for 72 h, cell pellets and culture media were collected and photographed. The melanin contents in cell pellets were further quantified, and melanin secretion was analyzed by measuring absorbance of culture media, as explained in the Materials and Methods section. Tyrosinase activity (**D**) and the protein (**E**) and mRNA (**F**) levels of melanogenesis-related genes were examined under identical conditions. As a positive control, arbutin was used. MITF: microphthalmia-associated transcription factor; MC1R: melanocortin 1 receptor; TRP: (tyrosinase-related protein). * *p* < 0.05; ** *p* < 0.01.

We first examined the viability of B16F10 melanoma cells cultured for 72 h in media made with 0, 25, 50, or 100% JMS. Significant cytotoxicity was observed in cultures grown in media containing 100% (*v/v*) JMS, but not 25 or 50% JMS, in the presence and absence of α-MSH (Figure 1A). Therefore, we used cell culture media containing up to 50% JMS in our subsequent studies. We observed that JMS significantly and time-dependently reduced the α-MSH-induced melanin content of the cultured cells (Figure 1B) and dose-dependently inhibited melanogenesis, as indicated by our analysis of melanin contents and secretion (Figure 1C). The activity of tyrosinase, which catalyzes the rate-limiting step in melanogenesis, was inhibited by JMS (Figure 1D). The protein (Figure 1E) and mRNA (Figure 1F) levels of the melanogenesis-related genes, TRP1 (tyrosinase-related protein-1), TRP2, tyrosinase, MITF, and MC1R, were also significantly decreased by JMS. The effects of 50% JMS on melanogenesis were comparable to those of arbutin, a well-known inhibitor of melanogenesis (Figure 1C–F). These data highlight the novel potential of JMS as an inhibitor of melanogenesis.

### 2.2. JMS Inhibits Melanogenesis via Activation of AMPK

**Figure 2:**
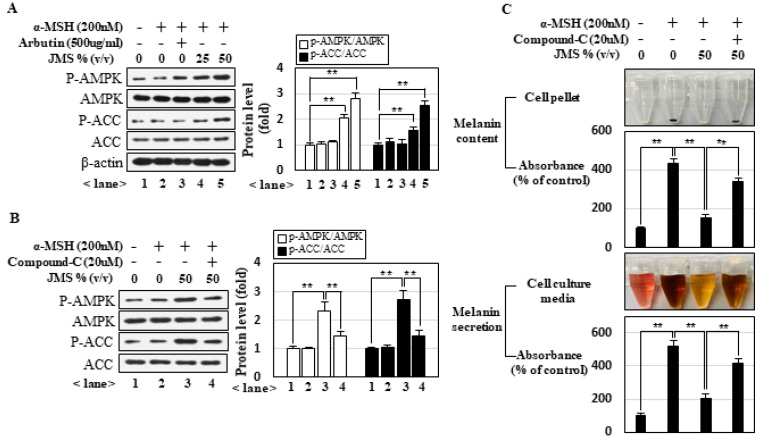

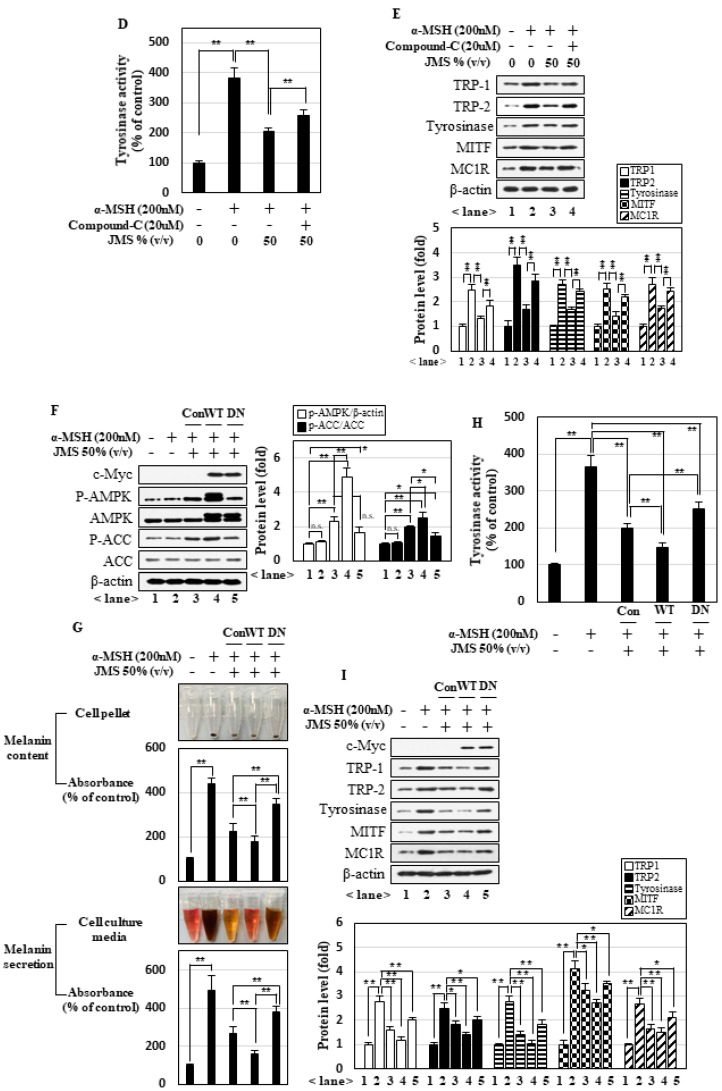
JMS inhibits α-MSH-induced melanogenesis via AMPK (5′ adenosine monophosphate-activated protein kinase) activation. B16F10 cells were treated with α-MSH and JMS in the absence or presence of the AMPK inhibitor, compound **C**, for 3 h (**A**,**B**) or 72 h (**C**–**E**). B16F10 cells were transfected with wild-type (WT) or dominant-negative AMPKα1 (DN) expression vector and then incubated with α-MSH and JMS (**F**–**I**). The cell lysates were subjected to Western blot analysis (**A**,**B**,**E**,**F**,**I**) and measurements of melanin contents, melanin secretion (**C**,**G**), and tyrosinase activity (**D**,**H**). ACC: acetyl-CoA carboxylase. * *p* < 0.05; ** *p* < 0.01.

Recent studies showed that AMPK mediates some of the beneficial effects of deep-sea water on several aspects of metabolism. However, little is known about the effects of JMS on AMPK or the potential role of AMPK in melanogenesis. Our results demonstrated that JMS activated AMPK in a dose-dependent manner, whereas α-MSH has no effect on AMPK, as judged by the phosphorylation level of Thr172 in AMPKα subunit and Ser79 of acetyl-CoA carboxylase, the substrate of AMPK (Figure 2A). To examine the possible role of AMPK in our system, we treated cells with the AMPK inhibitor, compound **C**, which effectively blocked JMS-induced AMPK activation (Figure 2B). The inhibitory effects of JMS on melanin contents, melanin secretion (Figure 2C), tyrosinase activity (Figure 2D), and the expression of melanogenesis-related genes (Figure 2E), were significantly attenuated in the presence of compound **C**. Although compound **C** has been extensively used in AMPK research, it exerts multiple side effects. Thus, we further demonstrated the role of AMPK was further demonstrated through the molecular approaches. Overexpression of c-Myc-tagged AMPKα1 wild-type (WT) potentiated JMS-induced AMPK activation, whereas the AMPKα1 dominant-negative (DN) form, in which the Thr172 residue was mutated to alanine, significantly blocked JMS-induced AMPK activation (Figure 2F). In accordance with the results obtained following the application of compound **C**, AMPKα1WT, but not AMPKα1DN, potentiated the inhibitory effects of JMS on melanin contents, melanin secretion (Figure 2G), tyrosinase activity (Figure 2H), and the expression of melanogenesis-related genes (Figure 2I). Collectively, our results suggest that AMPK mediates the inhibitory effects of JMS on melanogenesis.

**Figure 3:**
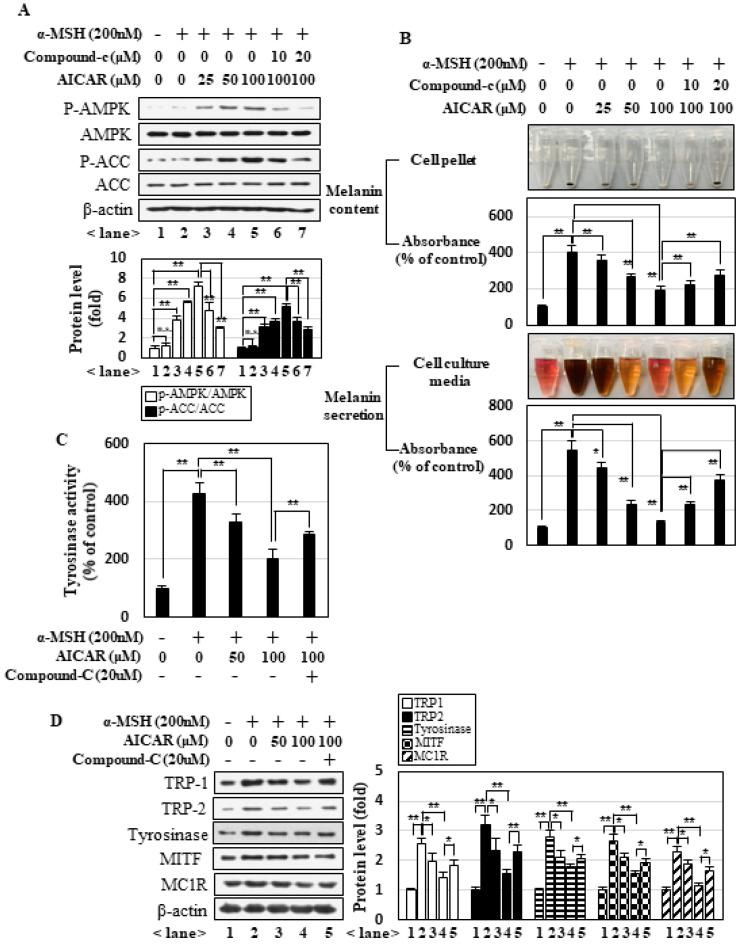

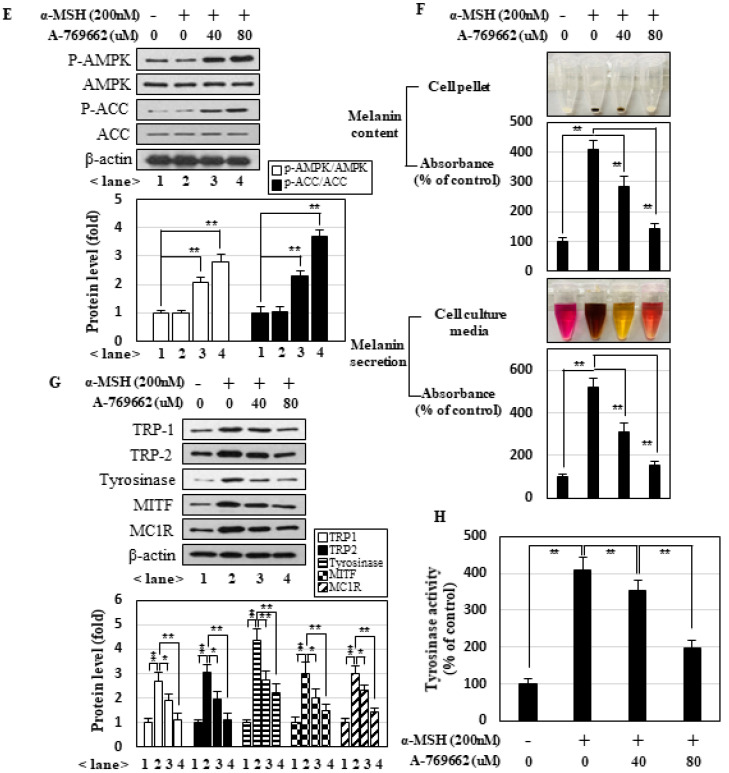
AMPK activation alone is sufficient to inhibit α-MSH-induced melanogenesis. B16F10 cells were treated with α-MSH plus the indicated concentrations of AICAR (aminoimidazole-4-carboxamide riboside), compound **C**, or A-769662 for 3 h (**A**,**E**) or 72 h (**B**–**D**,**F**–**H**). The cells were then subjected to Western blot analysis (**A**,**D**,**E**,**G**) and measurement of melanin contents, melanin secretion (**B**,**F**), and tyrosinase activity (**C**,**H**). * *p* < 0.05; ** *p* < 0.01.

Little work has been done investigating the possible role of AMPK in the regulation of melanogenesis. Although it is well known that AMPK mediates the anti-diabetic effects of metformin [33], a recent report demonstrated that metformin exerts anti-melanogenic effects independent of AMPK pathway [34]. In contrast, Kazinol U, a prenylated flavan extracted from dried root bark of Broussonetia kazinoki Sieb, was reported to inhibit melanogenesis via AMPK activation [35]. Thus, to unequivocally demonstrate the role of AMK in the regulation of melanogenesis, we investigated whether AMPK activation alone is sufficient to inhibit melanogenesis. Treatment of B16F10 melanoma cells with the cell-permeable AMPK activator, aminoimidazole-4-carboxamide riboside (AICAR), resulted in the dose-dependent activation of AMPK (Figure 3A) and concomitant suppression of the α-MSH-induced increases in the melanin contents, melanin secretion (Figure 3B), tyrosinase activity (Figure 3C), and expression of melanogenesis-related genes (Figure 3D). The effects of AICAR were significantly reduced in the presence of compound **C**. Since compound **C** has been shown to inhibit AICAR cellular uptake [36], we further examined the effects of a more selective agonist for AMPK, A-769662 [37], and the results showed that AMPK activation by A-769662 also resulted in suppression of melanogenesis (Figure 3E–H). These results suggest that AMPK activation is not only necessary to mediate the inhibitory effect of JMS, but also sufficient to inhibit melanogenesis.

### 2.3. JMS Activates AMPK in a CaMKKβ-Dependent Manner

**Figure 4:**
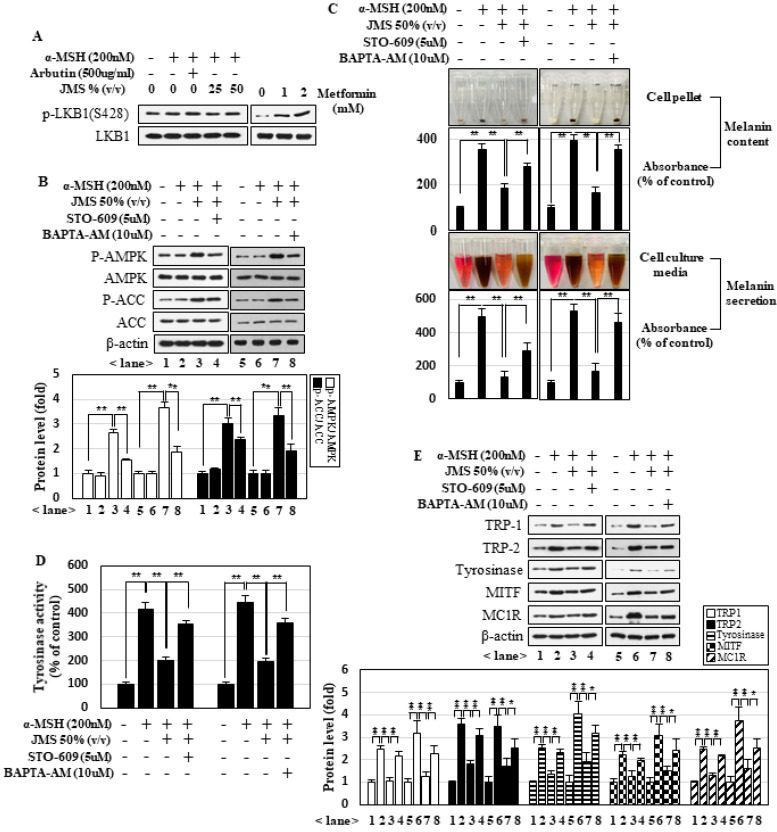
JMS inhibits α-MSH-induced melanogenesis via the CaMKKβ-AMPK signaling pathway. B16F10 cells were treated with α-MSH and JMS or metformin for 3 h (**A**) or for 24 h in the presence of STO-609 or BAPTA-AM (1,2-bis(o-aminophenoxy)ethane-N,N,N′,N′-tetraacetic acid) (**B**–**E**). The cells were then subjected to Western blot analysis (**A**,**B**,**E**) and measurement of melanin contents, melanin secretion (**C**), and tyrosinase activity (**D**). LKB1: liver kinase B1. * *p* < 0.05; ** *p* < 0.01.

To further understand the mechanism by which JMS activates AMPK, we examined the potential involvement of upstream kinases of AMPK. In addition to allosteric regulation, AMPK is phosphorylated at Thr172 in the α subunit and activated by LKB1 and Ca^2+^/calmodulin-dependent protein kinase-beta (CaMKKβ). Numerous studies reported that LKB1 is involved in activating AMPK under conditions characterized by changes in ATP and AMP, whereas CaMKKβ activates AMPK in a calcium-dependent manner [32]. Here, we first examined the phosphorylation level of Ser428 of LKB1 because this modification is known to be critical for AMPK activation in response to metformin treatment. Metformin did, indeed, increase the phosphorylation level of Ser428 of LKB1 in B16F10 cells under our treatment conditions; however, JMS treatment did not have any such effect (Figure 4A). In contrast, the ability of JMS to activate AMPK was significantly abrogated in the presence of the CaMKKβ inhibitor, STO-609, or a membrane permeable Ca^2+^ chelator, BAPTA-AM (1,2-bis(o-aminophenoxy)ethane-N,N,N′,N′-tetraacetic acid) (Figure 4B). The inhibitory effects of JMS on the melanin contents, melanin secretion (Figure 4C), tyrosinase activity (Figure 4D), and expression of melanogenesis-related genes (Figure 4E) were also significantly attenuated in the presence of STO-609 or BAPTA-AM. These data suggest that JMS suppresses α-MSH-induced melanogenesis via activation of the CaMKKβ-AMPK pathway.

### 2.4. JMS Inhibits Melanogenesis by Suppressing α-MSH-Induced PKA and MAPK Signaling

**Figure 5:**
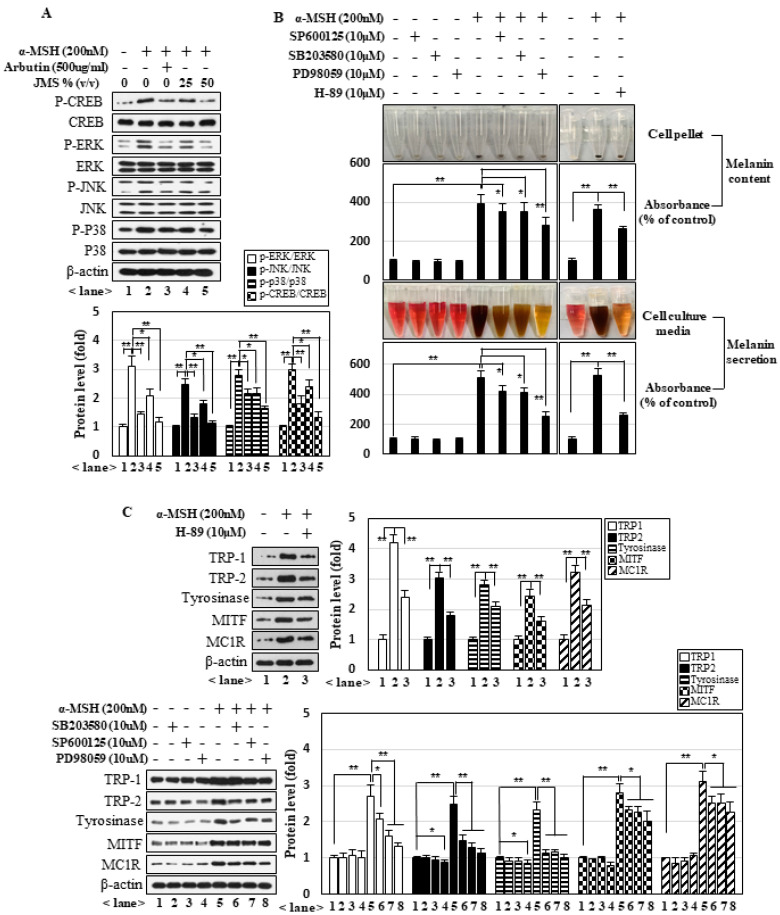
JMS inhibits the α-MSH-induced protein kinase A (PKA) and MAPK signaling pathways, which are critical for melanogenesis. B16F10 cells were treated with α-MSH and JMS for 3 h (**A**) or 24 h in the presence of inhibitors for MAPKs and PKA (**B**,**C**). The cell lysates were subjected to Western blot analysis (**A**,**C**) and measurement of melanin contents and melanin secretion (**B**). * *p* < 0.05; ** *p* < 0.01.

Many studies have suggested that PKA signaling is a major pathway leading to melanogenesis under α-MSH treatment. In addition, MAPK pathways have been implicated in α-MSH-induced melanogenesis. We observed that α-MSH activated the PKA and MAPK pathways in B16F10 cells, as judged by monitoring the phosphoactivated forms of CREB, ERK, JNK, and p38MAPK (Figure 5A). The application of H-89 (inhibitor of PKA), SP600125 (inhibitor of JNK), SB203582 (inhibitor of p38 MAPK), or PD98059 (inhibitor of ERK) significantly inhibited the ability of α-MSH to induce melanin contents, melanin secretion (Figure 5B), and the expression of melanogenesis-related genes (Figure 5C). JMS treatment inhibited the activation of these pathways in a concentration-dependent manner (Figure 5A). Together, these data suggest that JMS treatment inhibits the ability of α-MSH to activate the MAPK and PKA pathways, which are critical for melanogenesis.

### 2.5. JMS Treatment-Activated AMPK Inhibits α-MSH-Induced PKA and MAPK Signaling

**Figure 6:**
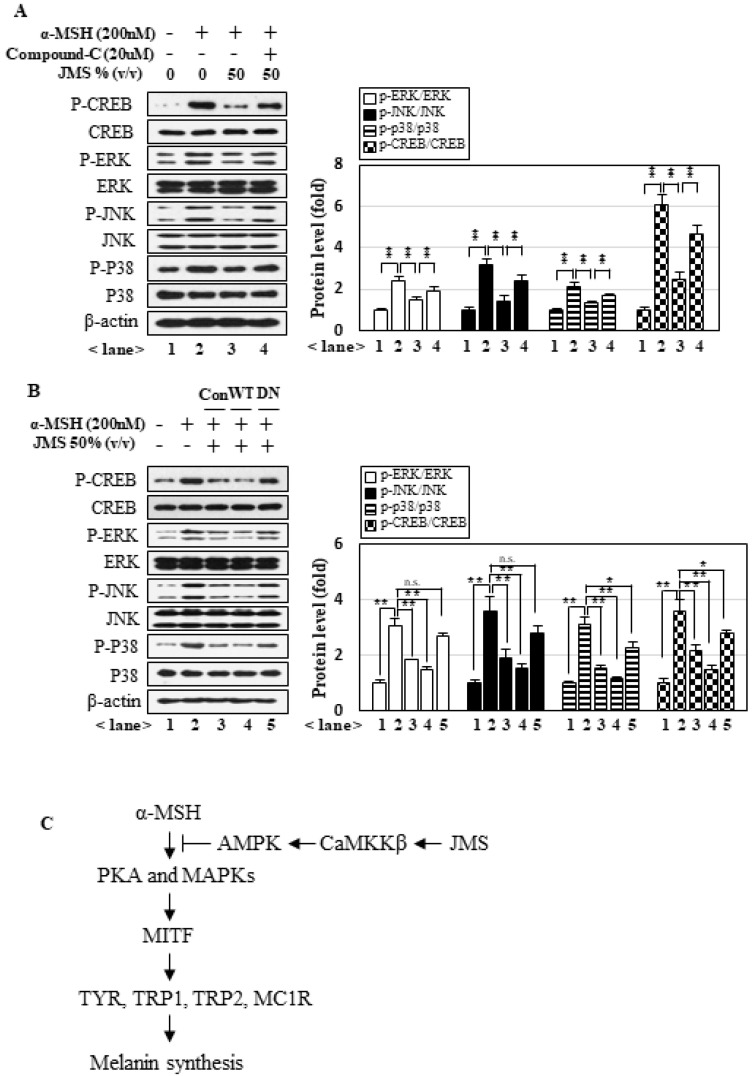
AMPK inhibits the α-MSH-induced MAPKs and PKA signaling pathways under JMS treatment. (**A**) B16F10 cells were treated with α-MSH and JMS for 3 h in the presence of compound **C**. (**B**) B16F10 cells overexpressing AMPKα1WT or DN were treated with α-MSH and JMS for 3 h. Western blot analysis was performed (**A**,**B**). (**C**) The signaling pathway proposed to be at work in B16F10 cells treated with α-MSH and JMS. * *p* < 0.05; ** *p* < 0.01.

We next investigated whether AMPK, which was highly activated by JMS treatment, interfered with downstream signal pathways of α-MSH. Indeed, we found that JMS treatment inhibited α-MSH-induced MAPK and PKA signaling, but these effects of JMS were significantly attenuated when AMPK was inhibited by the application of compound **C** (Figure 6A) or the expression of AMPKα1DN (Figure 6B). Collectively, our results suggest that JMS suppresses α-MSH-induced melanogenesis by activating AMPK, which interferes with the PKA and MAPK pathways.

## 3. Discussion

The human body requires minerals to maintain various essential physiological processes, and mineral deficiencies are tightly linked to numerous chronic disorders, such as type 2 diabetes, Alzheimer’s disease, hypertension, stroke, cancer, and cardiovascular disease [38,39,40,41,42]. Minerals such as calcium, zinc, copper, and magnesium also promote skin tissue growth and would healing [43,44,45,46]. However, we know little about how minerals affect melanogenesis. Recently, deep-sea water has been considered as a safe source of various minerals and has been reported to exert beneficial effects for the treatment of chronic diseases [20,21,22]. Deep-sea water, which refers to seawater pumped at a depth over 500 m, is typically cold, very pure, and rich in various minerals [22]. The mineral contents of desalinated underground water collected from Jeju Island of Korea (“JMS”) are similar to those of deep-sea water, and JMS has been reported to offer many of same beneficial effects against chronic diseases, including anti-obesity, anti-diabetes, and anti-steatotic effects [24,25,26]. The dietary intake of JMS was also reported to confer anti-inflammatory and anti-oxidant effects [26]. However, we know little about the potential effects of JMS on melanogenesis. In the present study, we report the potential of JMS as an anti-melanogenesis reagent and reveal the novel underlying mechanisms.

Our results suggest that α-MSH induces melanogenesis through the PKA and MAPK pathways, and that JMS inhibits melanogenesis via activation of CaMKKβ-AMPK pathways. These results are summarized in the diagram presented in Figure 6C. JMS contains various minerals that may contribute to activating AMPK, such as calcium. Our results showed that CaMKKβ, which calcium-dependently activates AMPK, appears to be the primary kinase that acts on AMPK in response to JMS treatment (Figure 4). Neurons and melanocytes stem from the neural crest, and CaMKKβ is a significant activator for AMPK in neurons [47]. Therefore, CaMKKβ seems to be a significant activator of AMPK in melanocytic cells. Our data indicate that the PKA and MAPK pathways play critical roles in α-MSH-induced melanogenesis (Figure 5), and that AMPK inhibits these pathways under JMS treatment (Figure 6). However, the direct targets of AMPK in these two pathways are essentially unknown. The interplay between the AMPK and PKA pathways seems to be highly complex and dependent on the context and experimental setting [48]. A recent study suggested that AMPK activation can inhibit PKA signaling by lowering the intracellular cAMP level. Treatment of hepatocytes with a small-molecule AMPK activator resulted in decrease in glucagon-stimulated cAMP accumulation, PKA activity, and downstream signaling, and AMPK was further revealed to directly activate cyclic nucleotide phosphodiesterase 4B (PDE4B), which converts cAMP to AMP [49]. In addition, AMP may play a dual role in the AMPK-mediated inhibition of the PKA pathway: it is critical for the allosteric activation of AMPK and also inhibits adenylate cyclase to block cAMP accumulation [50]. Although future work is needed for experimental validation, we speculate that, under JMS treatment, AMPK may inhibit the PKA pathway through a combination of the above-described pathways. Several reports found that AMPK inhibits hormone-induced ERK activation to suppress cell proliferations [51,52]. These results were interpreted as indicating that AMPK activation inhibits cell proliferation in response to a low cellular energy status, and thereby helps to conserve energy. In addition, a recent study showed that a selective MC4R (melanocortin 4 receptor) agonist suppressed neuroinflammation via the AMPK-dependent inhibition of the JNK and p38 signaling pathways [53]. However, the literature currently lacks a consensus regarding the interplay between AMPK and MAPKs; this is in large part because the activation of MAPKs results in a broad spectrum of physiological outcomes. In the present study, we add to this body of evidence by demonstrating that AMPK inhibits α-MSH-induced ERK, JNK, and p38MAPK signaling under JMS treatment. These results suggest that AMPK may regulate some point(s) common to these three MAPKs. 

There is a huge demand for melanogenesis inhibitors in the cosmetic industry and medicine. It is reported that approximately 15% of the world’s population invests in skin-whitening cosmetics, and global industry analysis forecasts predict that the global market for skin-whitening agents will reach $23 billion by 2020 [54]. Since tyrosinase catalyzes the rate-limiting step in melanogenesis, most of the pertinent research has focused on developing specific inhibitors against tyrosinase [55,56]. Indeed, most commercially available skin-lightening agents (e.g., arbutin, hydroquinone, kojic acid, and tranexamic acid) are tyrosinase inhibitors. However, these inhibitors suffer from certain drawbacks. Here, we demonstrate that JMS is highly safe and effective in inhibiting melanogenesis. We believe that JMS, which offers various pharmacological effects while being safe and inexpensive, may prove useful for a variety of purposes, such as in dietary supplements, pharmaceuticals, and cosmetics.

## 4. Materials and Methods 

### 4.1. Preparing Cell Culture with JMS

JMS was provided by Chunjieh Cooperation (Jeju City, Korea). It was pumped up from a depth of 130 m below sea level in the Han-Dong area of Jeju Island, Korea, and was desalinated by electrodialysis with 12 mS/cm conductivity. The composition and content of minerals in electrodialysis-desalinated JMS were previously reported [26]. Briefly Dulbecco’s modified Eagle’s medium (DMEM) powder was directly dissolved in 100% (*v/v*) desalinated JMS, and the pH was adjusted to 7.2 with bicarbonate. After filter sterilization, fetal bovine serum and antibiotics were added to 10% and 1%, respectively. The medium was further mixed with distilled water-based DMEM to obtain the desired percentage (*v/v*). The mouse B16F10 melanoma cell line was purchased from the American Type Culture Collection (Cat# CRL-6475) and maintained in DMEM with the indicated percentage of JMS at 37 °C in 95% air with 5% CO_2_.

### 4.2. Cell Viability Assay

B16F10 cells were seeded at 1 × 10^4^ cells/well in a 12-well cell culture plate and treated as indicated. After adding MTT (3-(4,5-dimethylthiazol-2-yl)-2,5-diphenyltetrazolium bromide) solution (500 μg/mL) to each well, the cells were incubated for 3 h at 37 °C. After removal of the MTT solution dimethyl sulfoxide (DMSO) was added, and the absorbance was measured at 570 nm using a microplate reader (BioTek, Winooski, VT, USA).

### 4.3. Stable cell Transfection

B16F10 melanoma cells were seeded on a six-well plate (Corning, NY, USA). When the cell density reached 50–60%, cells were transfected with the wild-type (WT) or dominant negative (DN, T172A) forms of c-Myc-tagged AMPKα1 expression vector for 24 h using Lipofectamine 3000 (Invitrogen, Carlsbad, CA, USA). Empty vector was used as a negative control. The cells were cultured for 7 days in the presence of G418 (1 mg/ mL), and the selected cells were used for the experiment.

### 4.4. Measurement of Melanin Contents and Secretion

Treated B16F10 melanoma cells were harvested and cell pellets were obtained and photographed. The pellets were dissolved in 1N NaOH containing 10% DMSO at 80 °C for 1 h, and the concentration of solubilized melanin was determined by measuring the absorbance at 405 nm using a microplate reader. To measure the degree of melanin secretion, culture media were collected after treatment, and absorbance was measured at 475 nm using a microplate reader. The melanin content and degree of melanin secretion were normalized to the cell number.

### 4.5. Intracellular Tyrosinase Activity

Treated B16F10 melanoma cells were lysed with the phosphate buffer saline (PBS) with 1% Triton X-100. Cell extracts were incubated with L-DOPA (2 mM) for 1 h at 37 °C, and the formation of dopachrome was determined by measuring the absorbance at 475 nm. 

### 4.6. Antibodies and Reagents

Antibodies against total LKB1(liver kinase B1) (3047), phospho (Serine 428)-LKB1 (3482), total AMPKα (5831), phospho (threonine 172)-AMPKα (2535), total ACC (3662), phospho (serine 79)-ACC (acetyl-CoA carboxylase) (3661), p44/42 MAPK (4695), Phospho-p44/42 MAPK (4376), p38 MAPK (9212), Phospho-p38 MAPK (9211), JNK (9252), Phospho-SAPK/JNK (4668), CREB (9197) and Phospho-CREB (9198) were purchased from Cell Signaling Technology (Danvers, MA, USA). Antibodies against tyrosinase related protein 1 (Trp1, sc-166857), tyrosinase related protein 2 (Trp2, sc-74439), tyrosinase (sc-20035), microphthalmia-associated transcription factor (MITF, sc-52938), β-actin (sc-47778), and c-Myc tag (sc-47694) were purchased from Santa Cruz Biotechnology (Dallas, TX, USA). The antibody against melanocortin 1 receptor (MC1R, PA5-21911) and all cell culture media and supplements were obtained from Thermo Fisher Scientific (Waltham, MA, USA) and Gibco (Grand Island, NY, USA). α-MSH, Arbutin, AICAR, compound **C**, PD98059, SP600125, SB203580, H89, A-769662, BAPTA-AM and STO-609 were obtained from Sigma-Aldrich (St. Louis, MO, USA).

### 4.7. Westernblot Analysis

Total proteins from B16F10 melanoma cells were extracted in RIPA (radioimmunoprecipitation assay) lysis buffer (50 mM Tris-HCl pH 7.4, 1 mM EDTA, 150 mM NaCl, 1% Triton X-100, 0.1% SDS, 0.5% sodium deoxycholate, 2 mM sodium orthovanadate, 5 mM sodium fluoride, 1 mM PMSF (phenylmethylsulfonyl fluoride), and a protein inhibitor cocktail). The protein concentration of the lysate was determined using the Bradford assay (Bio-Rad, Hercules, CA, USA). After separation of cellular proteins by sodium dodecyl sulfate-polyacrylamide gel electrophoresis (SDS-PAGE), proteins were transferred onto nitrocellulose membranes. The membranes were blocked with a blocking solution containing Tris-buffered saline with Tween 20 (0.1% Tween20, 10 mM Tris-HCl pH 7.4, 150 mM NaCl) and 3% bovine serum albumin (BSA), and incubated for 12 h at 4 °C with the appropriate primary antibody solution. After washing three times, the membranes were then incubated with the corresponding secondary antibody for 2 h at room temperature. The enhanced chemiluminescence (ECL) reagents were then applied.

### 4.8. Reverse Transcription-Polymerase Chain Reaction (RT-PCR)

Total RNA was extracted from B16F10 melanoma cells using the TRIzol reagent. Then, cDNA was made by reverse transcription of 1 µg total RNA using cDNA Synthesis Kit (Enzynomics, Daejeon, Korea). Following primers were used for PCR: TRP1, 5′-TCG AGA AGA ATG AAA TCT TA-3′ (forward) and 5′-AAG TGG CTC TTT TCT TCT GG-3′ (reverse); TRP2, 5′-TAC CAT CTG TTG TGG CTG GA-3’ (forward) and 5′-CAA GCT GTC GCA CAC AAT CT-3′ (reverse); tyrosinase, 5′-TCT TCA CCA TGC TTT TGT GG-3′ (forward) and 5′-ATA GGT GCA TTG GCT TCT GG-3′ (reverse); MITF, 5′-AAA CCA GCC TGG CGA CCA TGC C-3′ (forward) and 5′-TCA AGT TTC CAG AGA CGG GT-3′ (reverse); MC1R, 5′-TGC TGC CTG GCC CTG TCT GA-3′ (forward) and 5′-CGG ATG GAC CGC CGC CTT TT-3′ (reverse); β-actin, 5′-CTG TGC TGT CCC TGT ATG CCT C-3′ (forward) and 5′-CCA GAC AGC ACT GTG TTG GC-3′ (reverse). PCR was performed under the following thermo-cycling regimen: 2 min at 95 °C; 30 cycles of 95 °C for 40 s, 55 °C for 40 s, and 72 °C for 40 s; a final 2 min at 72 °C. The PCR products were analyzed by agarose electrophoresis, and the PCR data were normalized to the β-actin level.

### 4.9. Statistical Analysis

The experimental results are expressed as the mean ± standard error of the mean (SEM) obtained from at least three independent experiments. The statistical significance of the data was analyzed by one-way analysis of variance (ANOVA) using the R program suite (version 3.2.4; http://www.r-project.org). A *p*-value < 0.05 was considered statistically significant (* *p* < 0.05; ** *p* < 0.01).
